# Simultaneous Sizing and Refractive Index Analysis of Heterogeneous Nanoparticle Suspensions

**DOI:** 10.1021/acsnano.2c06883

**Published:** 2022-12-16

**Authors:** Unai Ortiz-Orruño, Romain Quidant, Niek F. van Hulst, Matz Liebel, Jaime Ortega Arroyo

**Affiliations:** † Nanophotonic Systems Laboratory, Department of Mechanical and Process Engineering, ETH Zurich, Zurich8092, Switzerland; ‡ ICFO, Institut de Ciencies Fotoniques, The Barcelona Institute of Science and Technology, Castelldefels08860, Spain; § ICREA, Institució Catalana de Recerca i Estudis Avançats, Barcelona08010, Spain

**Keywords:** nanoparticle tracking analysis, nanosizing, materials characterization, protein corona, holography

## Abstract

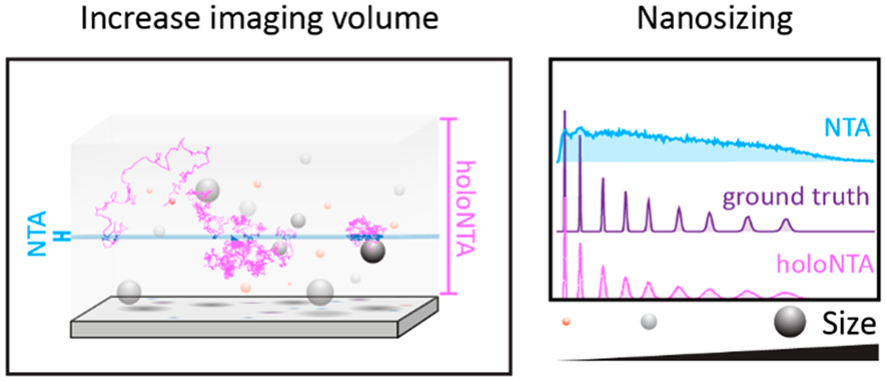

Rapid and reliable characterization of heterogeneous nanoparticle suspensions is a key technology across the nanosciences. Although approaches exist for homogeneous samples, they are often unsuitable for polydisperse suspensions, as particles of different sizes and compositions can lead to indistinguishable signals at the detector. Here, we introduce holographic nanoparticle tracking analysis, holoNTA, as a straightforward methodology that decouples size and material refractive index contributions. HoloNTA is applicable to any heterogeneous nanoparticle sample and has the sensitivity to measure the intrinsic heterogeneity of the sample. Specifically, we combined high dynamic range k-space imaging with holographic 3D single-particle tracking. This strategy enables long-term tracking by extending the imaging volume and delivers precise and accurate estimates of both scattering amplitude and diffusion coefficient of individual nanoparticles, from which particle refractive index and hydrodynamic size are determined. We specifically demonstrate, by simulations and experiments, that irrespective of localization uncertainty and size, the sizing sensitivity is improved as our extended detection volume yields considerably longer particle trajectories than previously reported by comparable technologies. As validation, we measured both homogeneous and heterogeneous suspensions of nanoparticles in the 40–250 nm size range and further monitored protein corona formation, where we identified subtle differences between the nanoparticle–protein complexes derived from avidin, bovine serum albumin, and streptavidin. We foresee that our approach will find many applications of both fundamental and applied nature where routine quantification and sizing of nanoparticles are required.

Label-free sensing and size-quantification methods are widely applied across the nanosciences and life sciences.^1−9^ These methods are key enablers for both fundamental research as well as day-to-day screening and quality-control applications. Among the many available techniques, all-optical approaches are especially promising due to their noninvasive nature and often straightforward and inexpensive experimental implementation. These methods can directly infer the size of an object via its scattering cross section^10^ or indirectly by, for example, analyzing particle-motion.^11−13^ Holographic or dark-field-type methods are prominent examples for the former class, and dynamic light scattering or nanoparticle tracking analysis (NTA) methods are examples for the latter.^14^ NTA infers the hydrodynamic diameter (*d*) of many individual micro- or nanoparticles (NPs) by following their Brownian motion over extended observation times. Albeit being incredibly powerful for larger NPs, with diameters of >100 nm, NTA and more recent holographic nanoparticle tracking implementations struggle with smaller NPs,^15−17^ which exhibit very large diffusion coefficients and small signals. These particles rapidly traverse the quasi two-dimensional observation plane of a conventional microscope within a few camera frames, which dramatically degrades the sizing precision. Solutions to the issue of finite track lengths have been explored in the form of high speed acquisitions,^18^ volume confinement via nanofluidic channels,^19^ or lock-on detection.^20^ However, in the absence of specialized hardware, holographic sensing-platforms with immobilized particles are often the only option for sizing small NPs. These methods are able to detect and quantify particles as small as single proteins in a label-free fashion, but estimate size purely based on scattering amplitudes (σ_S_) or particle-induced phase-changes.

Beyond the sensitivity aspects, accurately characterizing unknown particle populations remains a major limitation of all aforementioned techniques (Figure 1). Label-free methods rely on calibrations, a necessary step to correlate scattering amplitudes with particle size or to verify the measurements of Brownian motion-based NTA. Typical calibration-samples exhibit known dielectric constants (refractive indices, *n*_sample_), narrow size ranges (diameters, *d*_sample_), and similar surface-charge (*C*_sample_). A typical biomedical sample such as extracellular vesicles (EVs) can be very heterogeneous in size, exhibit widely varying compositions and hence particle-dependent refractive indices,^21^ and contain positively, negatively, and uncharged particles (Figure 1a). Similar particle populations are also ubiquitous in the field of nanomedicine with the synthesis and development of targeted drug delivery systems, and colloidal chemistry, where different nanoparticle populations are synthesized and their interaction with other reagents is tested, e.g., protein corona formation.^22^ These aspects lead to a number of complications. First, scattering amplitudes might be ill-suited for size-estimation (Figure 1b). Second, diffusion-based measurements are unable to identify dramatically different particles of similar size (Figure 1c), such as contaminations in the form of protein aggregates from EVs. Additionally, techniques that rely on nonspecific surface-binding of the analyte can be strongly biased, as they only capture a fraction of the, potentially, randomly charged sample.^23^ Furthermore, particle-dependent buoyant densities and size-dependent Brownian motion might further distort the surface-based particle capture, thus rendering quantitative concentration measurements difficult to impossible.

**Figure 1:**
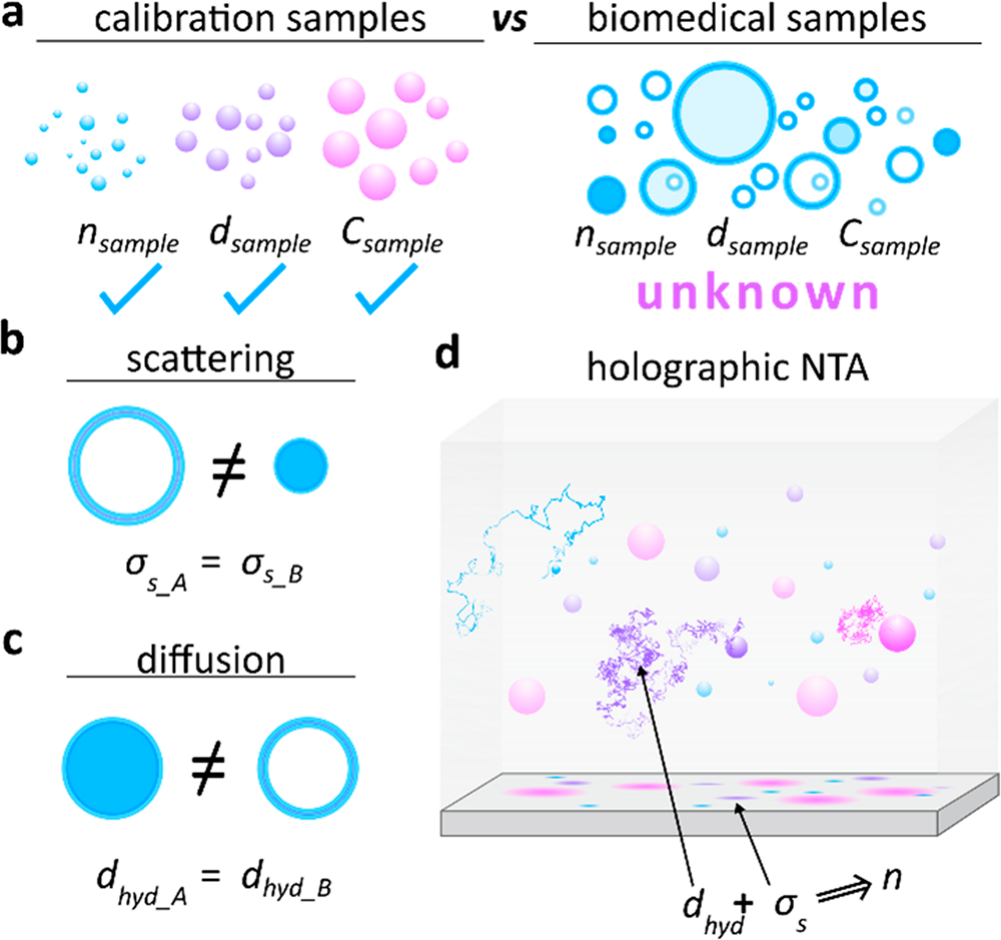
Challenges in nanoparticle sample characterization. (a) Typical calibration samples often do not reflect biomedical reality. The former exhibit well-defined dielectric constants, narrow size-ranges, and known surface charges. The latter are heterogeneous and composed of different biological building-blocks. (b) If the composition is unknown, particle size cannot be determined from scattering measurements. (c) Diffusion-based nanoparticle tracking analysis (NTA) measures particle size but cannot infer the material composition. (d) Holographic NTA simultaneously measures a particle’s hydrodynamic radius and scattering amplitude and furthermore eliminates the need for potentially biased surface-binding. Combined, these quantities allow inferring the dielectric constant of the particle and hence its biological composition.

In this work, we address the aforementioned challenges by implementing a holographic nanoparticle tracking analysis (holoNTA) platform for the precise and accurate characterization of nanometric samples. Our approach is schematically outlined in Figure 1d. We holographically observe a sample composed of freely diffusing particles to simultaneously measure the particles’ Brownian motion, as well as their scattering amplitudes. The approach allows estimating both the particles’ diameters, *d*, and refractive indices, with single particle sensitivity, thus yielding important information about their composition through refractive index characterization. Beyond measuring quantitative scattering amplitudes, holographic imaging enables digital refocusing which dramatically increases the volume-of-observation compared with the quasi 2D observation-plane of conventional NTA. The approximate 40-fold increase in the observed *z*-range allows recording long particle trajectories and confidently characterizing the diffusion coefficient of tiny NPs. This increase in trajectory lengths delivers the necessary size sensitivity to measure the intrinsic size dispersion of the nanoparticle population. With our experiment we furthermore have a 10× higher particle throughput compared to commercial NTA platforms. Ultimately, we rely on high dynamic range holography,^5^ via k-space imaging, to enable the simultaneous detection of particles with vastly different sizes.

## Results and Discussion

**Figure 2:**
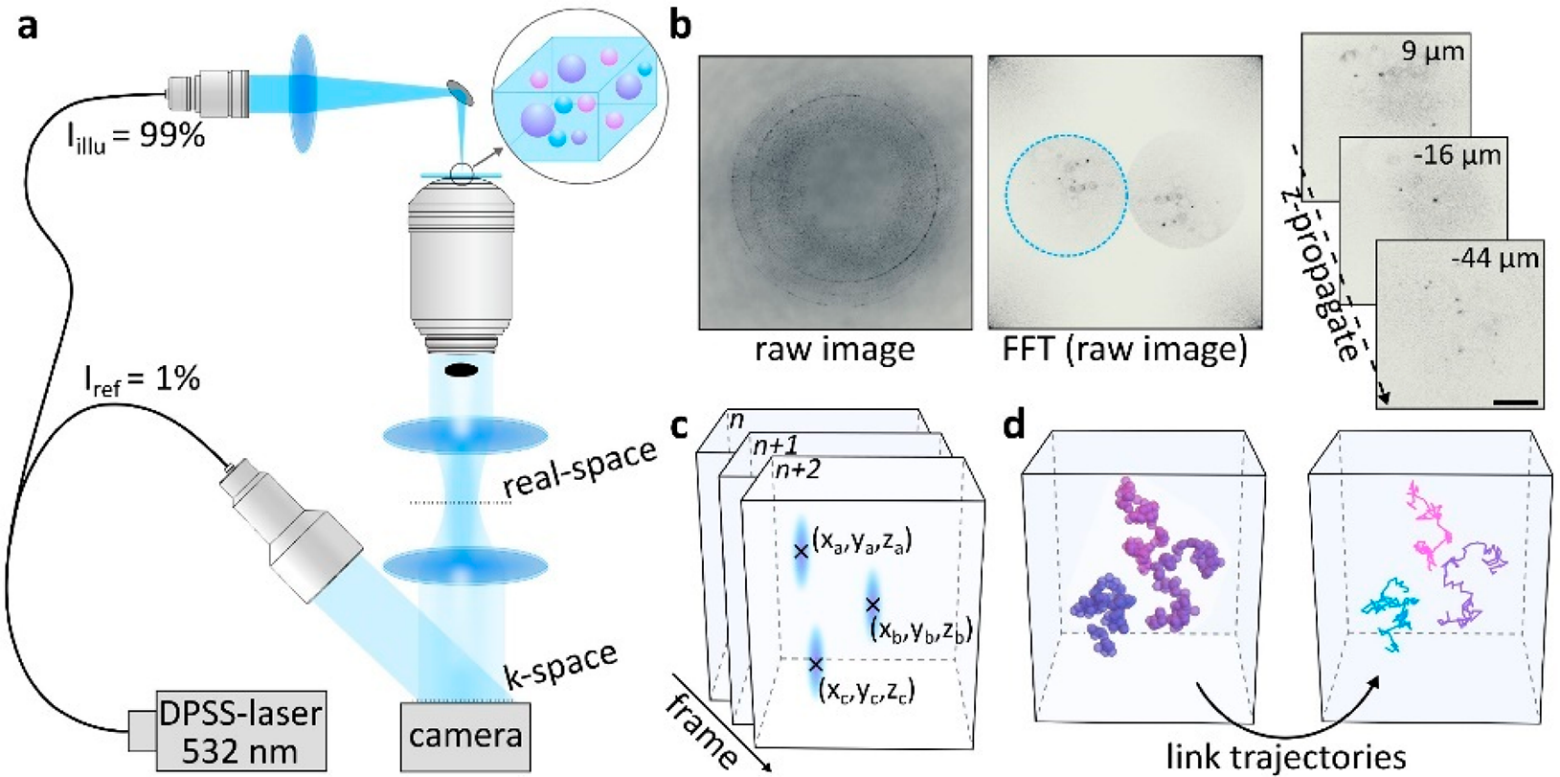
Sensor, data extraction, and processing. (a) Typical holographic NTA sensor based on a k-space holographic sensing platform. (b) Representative hologram alongside a Fourier filter extracted sample image, and examples of multiple sample-planes obtained after computational image propagation. Scale bar: 25 μm. All images are amplitude-capped for representation-purposes. (c) Analysis workflow based on 3D particle localization for all camera frames followed by (d) trajectory linking and mean-square-displacement analysis.

Figure 2a schematically depicts the holoNTA platform (Methods). A 99:1 fiber beamsplitter generates both the illumination (99%) and the reference wave (1%) for off-axis k-space holography. The former is focused onto the sample, mounted on top of an inverted microscope. The use of a long focal length lens ensures near-plane wave illumination of the sample volume, which is positioned within the Rayleigh range of the focusing wave. A dark-field mask, placed in the back-focal-plane of the microscope objective, selectively blocks the transmitted illumination light. Sample scattering passes the mask toward higher angles and is relay-imaged onto a camera where off-axis interference with the reference wave occurs. Figure 2b shows a representative hologram alongside its Fourier transformation where the interference term, reminiscent of a real-space image of the sample, is highlighted by a circle.

Following minor normalizations and phase adjustments to correctly center the back-focal-plane and remove residual wavefront curvature (Methods), we generate a 3D representation of the sparse sample by image propagation over a 50–80 μm *z*-range (Figure 2b, Methods). We then localize all particles within the digitally recovered large observation volume. Ultimately, we obtain position (*x*, *y*, *z*) and scattering amplitude estimates for all particles (Figure 2c). Repeating the workflow outlined above for videos composed of several hundred to thousands of frames yields Brownian motion trajectories for all particles and allows determining hydrodynamic diameters via their mean-square-displacement (MSD) curves (Figure 2d, Methods). Importantly, the simultaneous scattering amplitude measurement also enables inferring the particles’ composition, or refractive indices, if combined with a calibration based on particles with known compositions.

Compared to conventional NTA, holoNTA dramatically increases the sampled volume via numerical refocusing and hence the observation time before individual particles leave the detection window. Under Brownian motion, the relation between the time it takes for a particle to travel a certain distance, *x*_b_, e.g., the boundaries of the imaged volume, can be derived from the typical first passage time, *t*_p_, as *t*_p_ = *x*_b_^2^/(2*D*), where *D* is the diffusion coefficient of the particle.^24^ In practical terms, when the depth of focus is the limiting dimension in the imaged volume, holographic detection can routinely extend it by at least an order of magnitude. This in turn increases the total observation time of particles tracked by holoNTA by at least 2 orders of magnitude compared to NTA. In addition, this reduces the number of double-detected particles, a common problem in NTA, where particles leave and then return to the small volume-of-observation, which has been associated with issues in repeatability among different commercial implementations.^16^

The uncertainty, σ_D_/*D*, in determining the diffusion coefficient from the MSD curves, and thereby in sizing the nanoparticles relies on several factors: specifically, the overall trajectory length, *N*; the number of points used to fit the MSD curve; and the reduced localization error.^25,26^ The reduced localization error, χ, is expressed as χ = σ^2^/(*D*Δ*t*), and can be understood as the ratio between the static localization uncertainty and the displacement of the nanoparticle within consecutive frames. Specifically, σ and Δ*t*, correspond to the localization uncertainty and time between consecutive frames, respectively. For cases where χ ≪ 1 and recalling that σ_D_/*D* ∼ σ_d_/*d* by error propagation, the relation between the sizing uncertainty and the track length is given as  The above condition, χ ≪ 1, is satisfied for measurements with low localization uncertainty of particles with large diffusion coefficients, i.e., nanoparticles. It is important to note that this relation applies to the relative size uncertainty and not the absolute one; therefore, larger particles exhibit higher absolute dispersion values compared to smaller ones.

Combining the expressions of first passage time together with the relative size uncertainty as a function of track length, *N*, one can intuitively note that increasing the depth of focus by a factor, *m*, results in longer tracks by a factor of *m*^2^. This in turn reduces the relative size uncertainty by approximately this same factor, *m*, hence dramatically improving the accuracy and precision.

**Figure 3:**
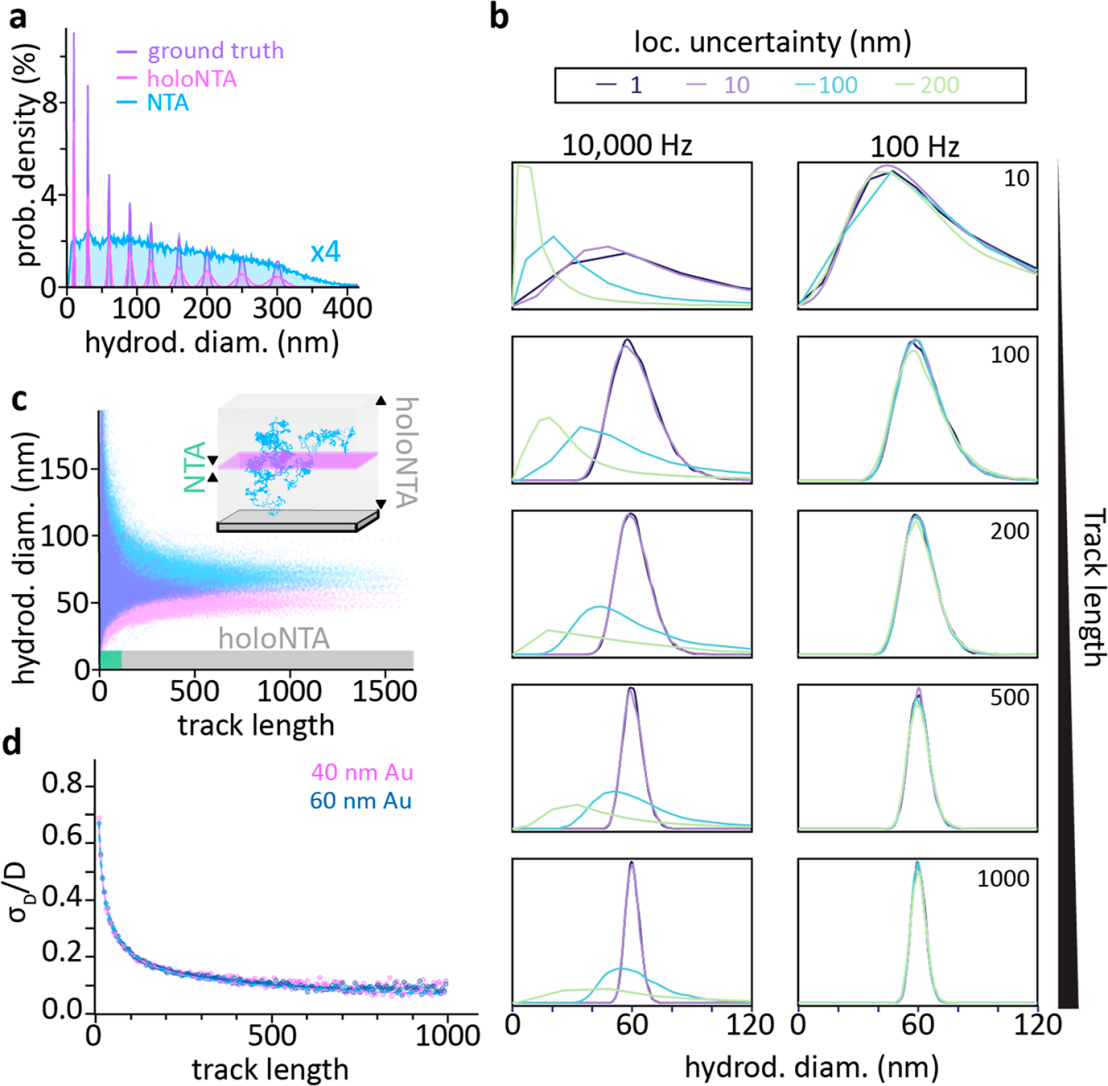
The advantage of holoNTA. (a) Simulation comparing ground-truth with NTA- and holoNTA-recovered size distributions. (b) Simulation comparing the effect of localization uncertainty, track length, and temporal resolution on the size distribution of nanoparticles. Track length increases from top to bottom. Different colors represent different localization uncertainty applied in each dimension. (c) Experimentally obtained track-length-dependent hydrodynamic diameters of samples containing either 40 or 60 nm Au NPs suspended in water (camera frame rate = 156 Hz, *t*_exp_ = 100 μs). Shaded regions represent typical lengths accessible to NTA (green) and holoNTA (gray). (d) Experimentally determined effect of track length on the relative uncertainty in determining the diffusion coefficient and thereby size for two different particle sizes. Solid lines show fit to equation of the form *Ax*^–0.5^ + *b*.

To put these abstract numbers into a more accessible form, we simulate NTA and holoNTA experiments for freely diffusing particles, assuming a heterogeneous sample composed of identical fractions of NPs with sizes ranging from 10 to 300 nm (Methods). To approximate a realistic experimental scenario, we assume 20 s image acquisition at 100 frames-per-second and a volume of observation of 80 × 80 × 4 μm^3^ (NTA) and 80 × 80 × 80 μm^3^ (holoNTA), respectively. Each NP diffuses from the center of this volume either until it leaves the observation volume or until 2000 frames have been recorded. Figure 3a compares the performance of the two methodologies with respect to the size estimate that would have been obtained if each NP would have been observed for 2000 frames, defined here as ground truth. While holoNTA only suffers a minor reduction in sizing-accuracy, NTA completely fails to recover the particle distribution of this heterogeneous sample.

The above simulation neglected the effect of localization uncertainty. Thus, we simulate the effect of both localization and track length to determine which of the two parameters has a stronger influence on particle size determination. For analysis, we take 10^4^ particles and vary the localization uncertainty in each dimension from 1 to 200 nm, and the track length from 10 to 1000 time points (Figure 3b). To match experimental conditions in terms of frame time, we set the time in between steps as 10 ms (100 Hz temporal resolution). Importantly the reduced localization error, χ, varies from much smaller than to approximately one within the probed parameter space. As such, the precision, i.e., dispersion in the size distribution, is independent of localization uncertainty, and instead is mainly determined by the track length, as expected from theory. In contrast, upon increasing the temporal resolution to 0.1 ms (10 000 Hz), analogous to approaches that rely on high sampling rates to obtain long tracks, the sizing distribution is sensitive to localization uncertainty, as the reduced localization error is no longer smaller than 1. These results emphasize a key advantage of holoNTA with respect to others; specifically, holoNTA minimizes the dependence on the localization uncertainty. To summarize, from a theoretical point of view, holoNTA robustly improves the accuracy and precision in size determination by extending the detection volume, thus effectively increasing the trajectory length.

To experimentally validate these theoretical predictions, we perform holoNTA measurements on samples composed of freely diffusing Au NPs with nominal diameters of either 40 or 60 nm. Figure 3c shows the experimentally obtained hydrodynamic diameter estimates as a function of the available track length used for extracting them. Figures 3d expresses these results in terms of the relative uncertainty in hydrodynamic diameter as a function of track length. Namely, the measurements closely follow the expected  scaling irrespective of particle size, up until the results converge to the inherent dispersion of the nanoparticle population. Importantly, the widths of the size distributions obtained for >1000 frames reflect the intrinsic 8% size-heterogeneity of the as purchased Au NP samples. In other words, holoNTA should be well-suited for accurately characterizing both homogeneous and heterogeneous samples with realistic size-distribution ranges.

**Figure 4:**
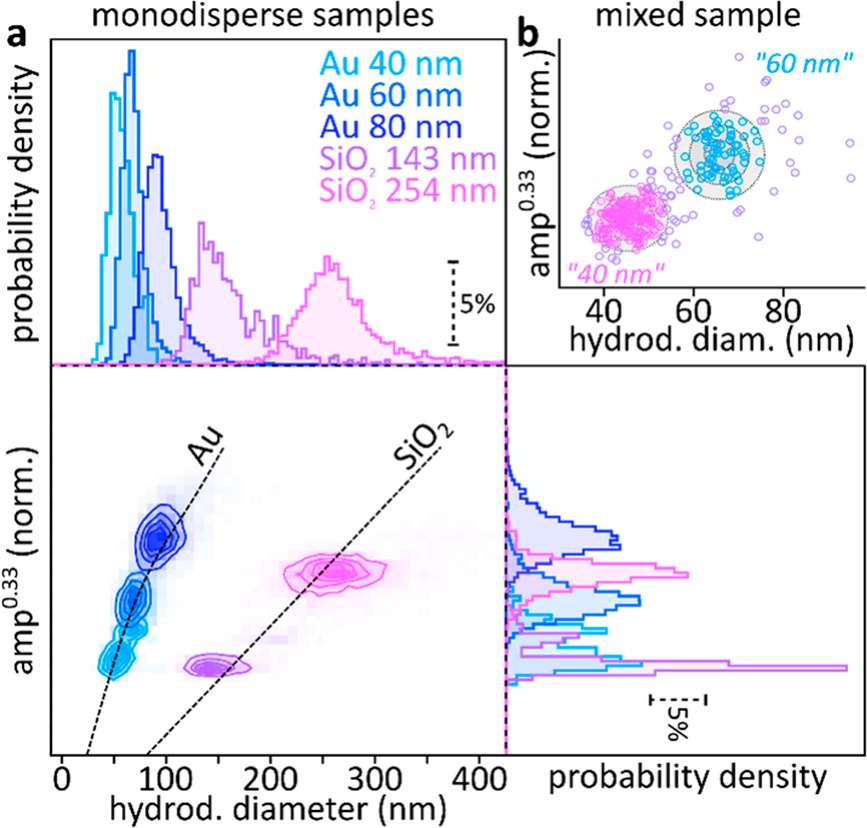
HoloNTA measurements of known samples. (a) Experimental results obtained for holoNTA measurements of aqueous NP dispersions for five different NP samples. Normalized cube-root amplitudes are shown to account for the diameter-cube dependence of scattering amplitudes. The dashed lines show Mie theory based scattering amplitudes for forward scattering of Au and SiO_2_ NPs at 532 nm in water. (b) Cube-root amplitude vs hydrodynamic diameter plot obtained for a mixed sample containing 40 and 60 nm Au NPs. The rings represent the result of a fit to a bimodal distribution (sum of two Gaussians) with the representative rings indicating confidence intervals of 1, 2, and 3 standard deviations. All measurements were performed using identical illumination intensities and camera integration times (*t*_exp_ = 100 μs, camera frame rate = 78 Hz). Number of particles tracked: (a) 320 (40 nm Au), 4394 (60 nm Au), 5249 (80 nm Au), 728 (143 nm SiO_2_), 3050 (254 nm SiO_2_); (b) 310.

Following the discussion of the holoNTA platform and its advantages over traditional NTA, we now move toward thoroughly characterizing its performance. To this end, we measure several monodisperse NP samples, e.g., NPs with a well-defined mean diameter and composition that exhibit synthesis-dependent sample heterogeneity around their mean (Methods). We specifically keep the particle concentrations below 1 × 10^9^ particles/mL to reduce speckle noise contributions, and thus maintain shot-noise limited performance. Coincidently, conventional NTA shares this upper concentration limit. Figure 4a shows the result obtained for both spherical Au (40, 60, and 80 nm) as well as SiO_2_ (143 and 254 nm) NPs. We obtain near-normally distributed diameter and scattering amplitude estimates for all five particles that are well-separated in the holoNTA-enabled 2D representation (Figure 4a). This simultaneous two-parameter estimate furthermore allows inferring the NP composition. A comparison between our measurements and Mie theory-based diameter–amplitude curves for the two materials shows remarkably good agreement (Figure 4a, dashed lines).

The results presented in Figure 4a are encouraging and suggest that holoNTA is well suited for directly inferring the size and dielectric constant of single NPs without being biased by surface interactions or arrival-at sensing surface problems. However, most techniques are capable of accurately sizing monomodally distributed NP samples but struggle with bi- or multimodal distributions. HoloNTA is distribution unbiased as it determines the sample-characteristics by interrogating many individual NPs. Figure 4b shows experimentally obtained results for a sample containing 40 and 60 nm Au NPs alongside a bimodal normal distribution-based particle classification, thus underlining that holoNTA is not limited to specific size distributions.

**Figure 5:**
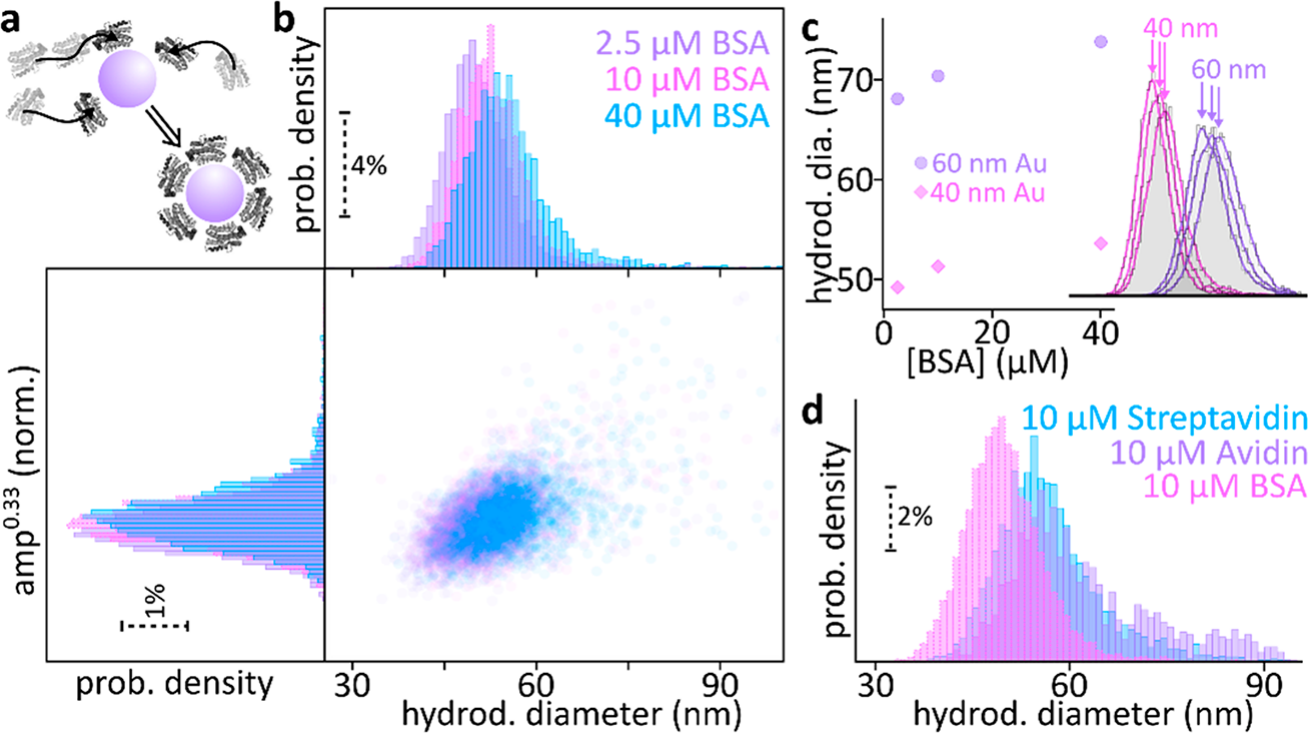
Sensing protein corona formation. (a) Upon contact with a biological fluid, particles are often coated in a so-called protein corona, a process that can dramatically alter the target specificity of tailor-made nanodrugs. (b) Holographic NTA reliably detects BSA protein corona formation around 40 nm diameter Au NPs. (c) The absolute hydrodynamic diameter changes of 40 and 60 nm Au NPs are near-identical. All standard deviations are <0.1 nm, and no error bars are shown. (d) Incubation with proteins of similar weight but different aspect ratio results in markedly different hydrodynamic diameter shifts. All measurements were performed using identical illumination intensities and camera integration times (camera frame rate = 156 Hz,; for each particle three measurements at integration times of *t*_exp_ = 20, 50, and 100 μs were combined). Number of particles tracked: (b, c) 6694 (2.5 μM), 6352 (10 μM), 5906 (40 μM); (d) 6352 (BSA), 1291 (avidin), 6352 (streptavidin). Hydrodynamic particle sizes (mean and standard error of the mean): (b, c) 2.5 μM (49.16 ± 0.05 nm, 68.02 ± 0.08 nm), 10 μM (51.36 ± 0.05 nm, 70.34 ± 0.08 nm), 40 μM (53.64 ± 0.08 nm, 73.73 ± 0.09); (d) BSA (51.36 ± 0.05 nm), avidin (57.1 ± 0.3 nm), streptavidin (54.91 ± 0.12 nm).

Following these validation experiments, we conclude with holoNTA measurements of protein corona formations around Au NPs (Figure 5a), an often-encountered problem when NPs come into contact with biological fluids. Due to the high protein concentrations in these fluids, the NPs are coated with a protein layer, a serious problem for nanomedical applications as surface-bound target information is easily masked. We choose such a model system because it is a highly relevant application to our platform, but more importantly, within the scope of this work, it allows us to evaluate the sensitivity of holoNTA to detect minute changes in hydrodynamic size. Figure 5b shows holoNTA measurements of the corona-formation process for 40 nm Au NPs incubated with different aqueous suspensions of bovine serum albumin (BSA) proteins. In agreement with our expectations, we observe an increase in hydrodynamic diameter with increasing BSA concentration.

To ensure that the hydrodynamic diameter change is not due to slight changes in fluid viscosity at increasing BSA concentrations, we compare the diameter changes for 40 and 60 nm Au NPs with respect to the relative viscosities changes as determined from the Krieger–Dougherty model.^27^ Using this model, within the measured BSA concentrations, the maximum change in viscosity is less than 0.8%. In contrast,Figure 5c shows that both particles undergo similar absolute diameter increases, reminiscent of adsorption of a protein layer of identical thickness, and with relative diameter changes of 9% (40 nm AuNPs) and 8% (60 nm AuNPs), respectively. Thereby we rule out changes in viscosity as the governing parameter in the observed particle size changes.

Finally, we explore hydrodynamic diameter changes for different proteins (BSA, avidin, and streptavidin) with similar molecular weights but varying aspect ratio and surface charge. Considering the isolectric point (pI) of the proteins, in solution BSA (pI = 4.7) and streptavidin (pI = 5.0) are negatively charged, whereas avidin (pI = 10.5) is positively charged. Despite the difference in surface charge, the current hypothesis is that the binding, and thus corona complex formation with negatively charged AuNPs is driven by the electrostatic interactions between the AuNPs and positively charged amino acids present in all three proteins: lysine, arginine, and histidine.^28−30^Figure 5d shows that all three proteins form coronas around the AuNPs. Furthermore, despite identical incubation times and protein concentrations, the resulting nanoparticle protein corona complexes exhibit noticeably different hydrodynamic diameters even though the three proteins exhibit similar molecular weights. BSA, in spite of being slightly heavier in molecular weight (66 kDa) than streptavidin (55 kDa) and similar to avidin (67–68 kDa), forms the smallest protein corona complexes. We hypothesize that the mean population difference in size between streptavidin and avidin protein corona complexes can be attributed to differences in molecular weight, whereas the existence of higher degree of dispersion for avidin complexes can be attributed to differences in protein surface charge. Namely, avidin molecules are more susceptible to nonspecific interactions with the negatively charged AuNPs,^31^ thus leading to larger protein corona complexes. More detailed studies are required to characterize the type of protein–nanoparticles interactions, such as protein layer density or protein geometry. Specifically *in situ* single particle binding kinetics studies,^20^ for which holoNTA is ideally suited, offer a promising route to obtain this information.

## Conclusions

To summarize, we experimentally implemented holographic nanoparticle tracking analysis (holoNTA), a much-needed extension of the extremely powerful and ubiquitously applied NTA methodology. HoloNTA simultaneously performs single-particle tracking and quantitative scattering measurements of freely diffusing (nano)particles. As a holographic technique, holoNTA readily accesses large 3D volumes-of-observation via digital hologram postprocessing, which enables long-term observations for accurate and precise NP sizing. Although holoNTA does require slightly sparser samples compared to NTA, it maximizes the data extraction from the measurements by at least an order of magnitude due to the increased volume sampled. We highlighted the capabilities of holoNTA by measuring both monodisperse and mixed nanoparticle samples and observing protein corona formation around small NPs. Ultimately, holoNTA allows accessing the dielectric constant of individual NPs, if precalibrated with a known reference material. These capabilities are comparable to the 2D analysis in flow cytometry that uses forward vs side scattering as an extremely powerful approach for single cell and even extracellular vesicle analysis.^21^

Conceptually, this work highlights a route to enhance the sizing precision by extending the length of individual particle trajectories. Specifically, we demonstrate this capability by increasing the total imaging volume by at least an order of magnitude compared to conventional NTA. As an alternative route, similar enhanced nanoparticle sizing performances have recently been achieved by either recording small imaging volumes at very high acquisition frame rates^18^ or by combining high acquisition frame rates with 1D confinement inside a nanochannel.^19^ It is important to remark that neither of these routes are mutually exclusive, i.e., large imaging volumes and high frame rate acquisition, and the main difference between them is how the track length increase scales with respect to the approach. In the case of volume extension, the average increase in trajectory length scales quadratically, whereas improving the temporal resolution does so linearly. In principle, holoNTA is entirely compatible with high temporal resolution. Despite having an overall temporal resolution on the order of 10 ms, as determined by the frame time of the camera, all experiments were performed with sensor integration times of 20–100 μs, analogous to Kashkanova et al.^18^ and Špačková et al.,^19^ which directly translates to >10 kHz frame rates. Our approach uses off-the-shelf industrial sensors and does not require nanofabrication, and as such we envision direct applications of our methodology for routine quantification and sizing in diverse fields of both fundamental and applied nature, ranging from heterogeneous catalysis over fundamental biology into the clinic.

## Methods

### Microscope

The microscope is a simplified design of our previously published k-scope.^5^ Both illumination and reference waves are obtained from a 532 nm DPSS laser (CW532-100, Roithner Lasertechnik) coupled into a 99:1 fiber beamsplitter (TN532R1A1, Thorlabs). The 99% fraction illuminates the sample in a transmission configuration, and sample scattering is collected by a water immersion objective and residual illumination light rejected via a dark-field mask placed into the back-focal-plane of the objective (UPLSAPO60XW/1.20, Olympus). A 0.5× relay imaging system forms an image of the objective’s back-focal-plane on the camera (a2A1920-160umBAS, Basler) where it interferes with the collimated reference wave in an off-axis configuration. The imaged k-space hologram corresponds to a camera sensor area of 1200 pixels × 1200 pixels. The optical path lengths of both waves are coarsely matched, within a few centimeters, to ensure optimal interference contrast. The size of the illumination beam impinging onto the sample, defined as the area encompassing >10% of the maximum amplitude, was measured experimentally and corresponds to a diameter of approximately 80 μm.

### Optical Imaging

For all experiments, 10 measurements corresponding to different locations in the sample were taken for each exposure time (*t*_exp_ = 20, 50, 100 μs). Each acquisition consisted of 2048 holograms recorded at either a camera frame rate of 78 Hz (Figure 4) or 156 Hz (Figure 5), corresponding to a recording time of about 26 and 13 s, respectively. The maximum frame rate of acquisition, 156 Hz, is limited by the minimum readout time of the camera. In all experiments, the sample is illuminated with 32 mW (fluence, 6.8 μW/μm^2^) and 18 μW is used as the reference intensity. Minor fluctuations between measurements are normalized by recording the power prior to each experiment.

### Hologram Processing

Prior to performing any data processing, we subtract the camera’s dark offset from all recorded images. For each experiment, we separately acquire an image of the reference wave, denoted here as reference, by blocking the sample illumination. We next subtract the reference from all acquired holograms and then divide the difference by the square root of the reference, thereby correcting for potential amplitude inhomogeneities. Inverse Fourier transforming the processed k-space holograms then reveals three nonoverlapping regions in image space: the real, twin, and zero-order images. We isolate the real image, corresponding to one of the interference terms, by Fourier filtering, which involves hard-aperture selection followed by phase shifting. Finally, the as-processed real image is Fourier transformed to yield the complex valued electric field in k-space which is subsequently used for further downstream processing.

### Background and Aberration Correction

To eliminate contributions from static scattering signals intrinsic to both the sample and to imperfections in the optical system, we generate a background based on the temporal median of the processed complex-valued k-space image and subtract it from all k-space images. We further account for the spatially nonuniform illumination profile, which affects the scattering signals. We reconstruct the beam profile based on the amplitudes and positions of all localized particles in 3D as previously reported.^5,32^ In short, we generate an image containing all localized particles, normalized to the number of detection events per position and then low-pass-filter this image. The resulting beam-area estimate is then normalized to unity and used to normalize all particle amplitudes at their respective *x*/*y*-position. Particles in the low-amplitude regions of the illumination profile, <10% of the maximum amplitude, are excluded from the analysis. To remove optical aberrations, we ensemble-average the normalized complex valued point spread functions of all particles from a representative video and isolate them from the rest of the ensemble image using a binary mask with a width of 10 times Nyquist. The resulting ensemble PSF image in real space is Fourier transformed, and the phase from the complex BFP image retrieved. Finally, we remove optical aberrations by deconvolving the real space images.

### Hologram Propagation and 3D Single Particle Tracking

The aberration corrected holograms are propagated along the optical axis according to the angular spectrum method. Specifically, the processed *M × M* pixel^2^ k-space holograms are multiplied by the propagation kernel **K** and subsequently inverse Fourier transformed. Specifically, the propagation kernel has the form
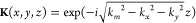
where *k*_*m*_ = 2π*n*/λ, with *n* being the refractive index of medium through which the light propagates, corresponding to water in this work. The discretized spatial frequencies are (*k*_*x*_, *k*_*y*_) = 2π(*x,y*)/(*M*Δ*x*) for (−*M*/2 ≤ *x*, *y* ≤ *M*/2) and with Δ*x* representing the magnified pixel size of the imaging system. For 3D localization, each hologram is first propagated from −40 μm up to +40 μm with a coarse spacing between different *Z*-planes (Δ*z*) of 400 nm. The resulting 3D intensity maps are then segmented into regions of interest based on local maxima. To achieve subpixel localization, the particle-containing segmented regions of interest are propagated with a finer Δ*z* spacing of 100 nm over a total of ±2 μm with respect to their local maxima. We then determine the particles’ maxima within a 1 μm × 1 μm region around the center-of-mass and subsequently fit a parabola using the two most adjacent *Z*-pixel values along the maximum. For subpixel localization along the *XY*-plane, particles that are in focus at the calculated *Z*-plane are fitted using the radial center symmetry algorithm.^33^ Finally, we follow the adaptive tracking algorithm of Jaqaman et al. to link all the 3D localizations and generate 3D tracks.^34^ Only tracks longer than 100 time points are used for further analysis.

### Trajectory Analysis

The size of each individually tracked particle is determined from the 3D MSD curve. In brief, we extract the diffusion coefficient from the slope of a linear fit of the first three points (*t* = *p*Δ*t* for *p* = 1, 2, 3) of the MSD following the expression MSD(*t*) = *x*_0_^2^ + 6*Dt.* We choose to only fit the first three points of the MSD curve as our experimental conditions closely satisfy the regime where χ ≪ 1, where χ = σ^2^/(*D*Δ*t*).^26^ Namely, in our experiments the value for  ranges from 100 to 270 nm, depending on the particle diameter (40–300 nm), whereas our static localization uncertainty, σ, is on the order of 10 nm (Supporting Information), making the reduced localization error χ < 0.01.

### Freely Diffusing Particles

First, a cover slide is placed on the sample holder of the microscope and a drop of particle solution is added. A second cover slide, mounted above the former, is then lowered onto the sample solution until contact. If necessary, we tilt-correct the orientation of the top cover slide to ensure that the back-reflections and the illumination wave are blocked by the dark-field mask. Following the sample-mounting we wait a few minutes to ensure that disturbances, such as internal flows, do not distort the measurements.

### Particle Solutions

Stock solutions of citrate-capped gold (40, 60 and 80 nm, BBI solutions) and silica (143 and 254 nm, microParticles GmBH) particles are diluted in water to the desired concentration of 2.5 × 10^8^ NP/mL.

### Protein Capped GNPs

BSA (A9418, Sigma-Aldrich), avidin (A9275, Sigma-Aldrich), and streptavidin (S4762, Sigma-Aldrich) are diluted in water to twice the desired final protein concentrations. Previously diluted nanoparticle dispersions are then mixed at a 1:1 ratio with the respective protein dispersions and left to incubate overnight for a final NP concentration of 1.25 × 10^8^ NP/mL.

### Diffusion Simulations

Monte Carlo simulation parameters for Figure 3a are the following: 100 frames per second, Brownian motion in water, maximum 2000 frames, NTA volume 80 × 80 × 4 μm^3^, holoNTA volume 80 × 80 × 80 μm^3^, 10 nm localization error (in each dimension). The particle is eliminated once it leaves the observation volume. The sample is composed of 10 000 NPs each with diameters of 10, 30, 60, 90, 120, 160, 200, 250, and 300 nm.

For Monte Carlo simulation parameters for Figure 3b, we model 10^4^ particles with nominal diameters of 60 nm undergoing Brownian motion. Here we vary the localization uncertainty in each dimension from 1 to 200 nm, and the track length from 10 to 1000 time points, and compute all possible combinations. To match experimental conditions, we set the time between steps (frame time/temporal resolution) as 10 ms. We then reconstruct the trajectories, apply the MSD analysis as detailed before, and determine the size distribution. For comparison with a faster acquisition scenario, we repeat the process but with a time between steps (temporal resolution) of 0.10 ms.

## Supporting Information Available

Characterization of the effect of localization uncertainty on nanoparticle sizing (Figure S1) and detailed experimental and data analysis workflows (Figures S2–S4) (PDF)
