# Supplementary material for: Simultaneous Sizing and Refractive Index Analysis of Heterogeneous Nanoparticle Suspensions
Source: ACS Nano. 2022 Dec 16;17(1):221–9. doi: 10.1021/acsnano.2c06883 (PMC9835976; doi:10.1021/acsnano.2c06883)
Supplement: Supplementary file 1 — nn2c06883_si_001.pdf [file nn2c06883_si_001.pdf]

SUPPLEMENTARY INFORMATION

Simultaneous sizing and refractive index analysis of heterogenous nanoparticle suspensions

Unai Ortiz-Orruño[2], Romain Quidant[1], Niek F. van Hulst[2,3], Matz Liebel[2,*] and Jaime Ortega Arroyo[1,*]

AFFILIATIONS:

[1] Nanophotonic Systems Laboratory, Department of Mechanical and Process Engineering, ETH Zurich, Zurich, 8092, Switzerland

[2] ICFO, Institut de Ciencies Fotoniques, The Barcelona Institute of Science and Technology, Castelldefels 08860, Spain

[3] ICREA, Institució Catalana de Recerca i Estudis Avançats, Barcelona, 08010, Spain

[*]Corresponding authors. Email: jarroyo@ethz.ch, matz.liebel@icfo.eu

Effect of localisation uncertainty on sizing

Following the same acquisition conditions as all other experiments (exposure time: 0.1 ms, frame rate: 156 Hz, fluence: 5.7 µW/µm^2, 10 different recordings of 13 s each) we benchmarked the effect of localisation uncertainty on the particle sizing using immobilised 80 nm Au particles. The results are summarised in Figure S1 below, where we specifically show a representative real-space image of the processed k-space hologram, a subset of particle trajectories and the corresponding fluctuations around the mean position in all three dimensions of a single particle. The analysis yields localisation uncertainties in all three dimensions on the scale of tens of nanometres.

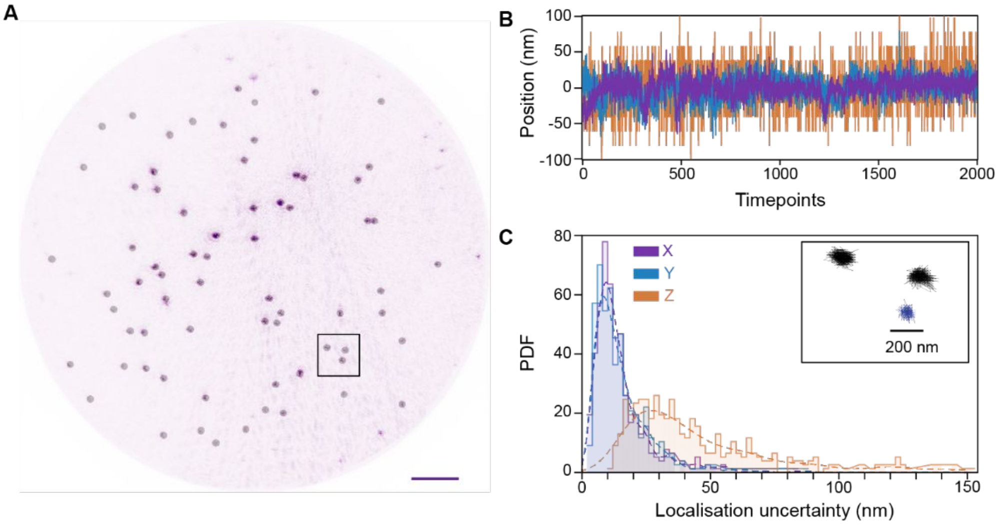

Figure S1: **Static localisation uncertainty benchmark**. (A) Representative real-space amplitude image of 80 nm Au particles immobilised on a glass substrate immersed in an aqueous solution. Gray overlaid circles mark the localised particles (B) Position time trace of a single Au particle, marked in blue in C, after subtraction of the mean. (C) Static localisation uncertainty distribution for an ensemble of AuNPs (N=326). Inset: 2D trajectories of the zoom-in section marked in A. The distances between particles are not to scale. Scale bar: 10 µm.

Detailed experimental and data analysis workflow

To obtain optical aberration-free holograms, we perform a data processing similar to the one described in (Ortiz-Orruño et al., 2021). First, prior to performing any data processing, we subtract the camera's dark-offset from all recorded images. For each experiment, we separately acquire an image of the reference wave, denoted here as reference, by blocking the sample illumination. We next subtract the reference from all acquired holograms and then divide the difference by the square root of the reference, thereby correcting for potential amplitude inhomogeneities, as described in Figure S2. Inverse Fourier transforming the processed *k*-space holograms then reveals three non-overlapping regions in image-space: the real, twin, and zero-order images, respectively (Figure 2b). We isolate the real image, corresponding to one of the interference terms, by Fourier filtering, which involves hard-aperture selection followed by phase shifting. Finally, the as-processed real image is Fourier transformed to yield the complex valued electric field in *k*-space which is subsequently used for further downstream processing.

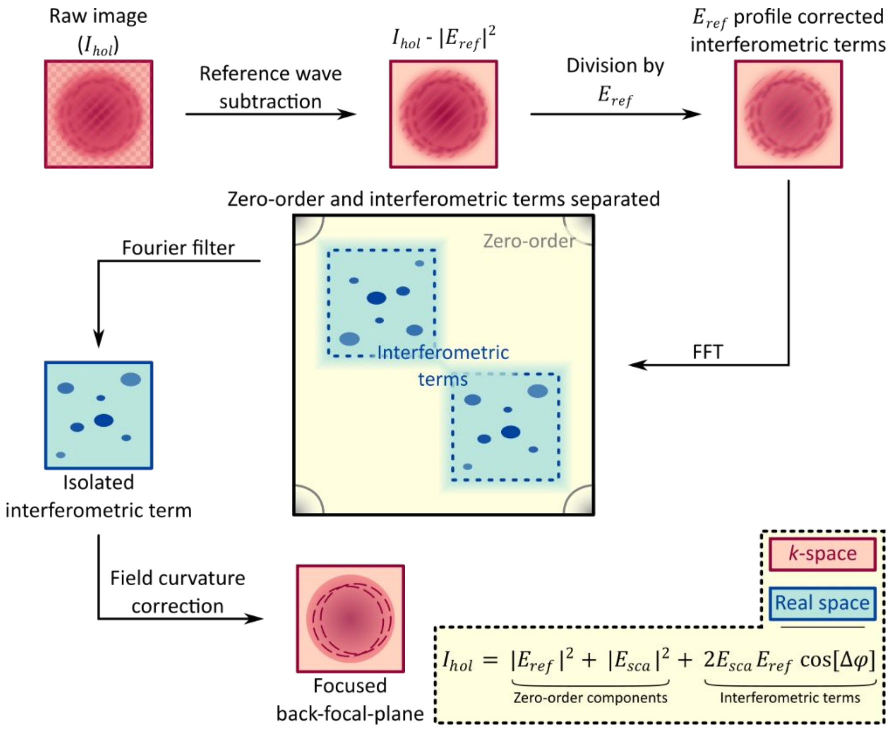

Figure S2, **Hologram processing.** Reference is subtracted from the raw data and the resulting image is divided further by the square-root of the reference. Signal is Fourier transformed, resulting in real-space representations of the interferometric terms separated from the zero-order component. One of the former representations is isolated by cropping the image and the field curvature corrected. Salmon colour refers to k-space while turquoise indicates real space.

To eliminate contributions from static scattering signals intrinsic to the sample and to imperfections in the optical system, we generate a background based on the temporal median of the processed complex-valued *k*-space image and subtract it from all *k*-space images (Figure S3). We further account for the spatially nonuniform illumination profile, which affects the scattering signals. We reconstruct the beam profile based on the amplitudes and positions of all localised particles in 3D, as described in Figure S3. In short, we generate an image containing all localised particles, normalised to the number of detection events per position and then lowpass filter this image. The resulting beam-area estimate is then normalized to unity and used to normalise all particle-amplitudes at their respective x/y-position. Particles in the low-amplitude regions of the illumination profile, <10% of the maximum amplitude, are excluded from the analysis. Figure S3 also shows how optical aberrations are removed. We ensemble average the normalised complex valued point spread functions of all particles from a representative video and isolate them from the rest of the ensemble image using a binary mask with a width of ten times Nyquist. The resulting ensemble point spread function image in real space is

Fourier transformed, and the phase from the complex back-focal-plane image retrieved. Finally, we remove aberrations by deconvolving the real space images.

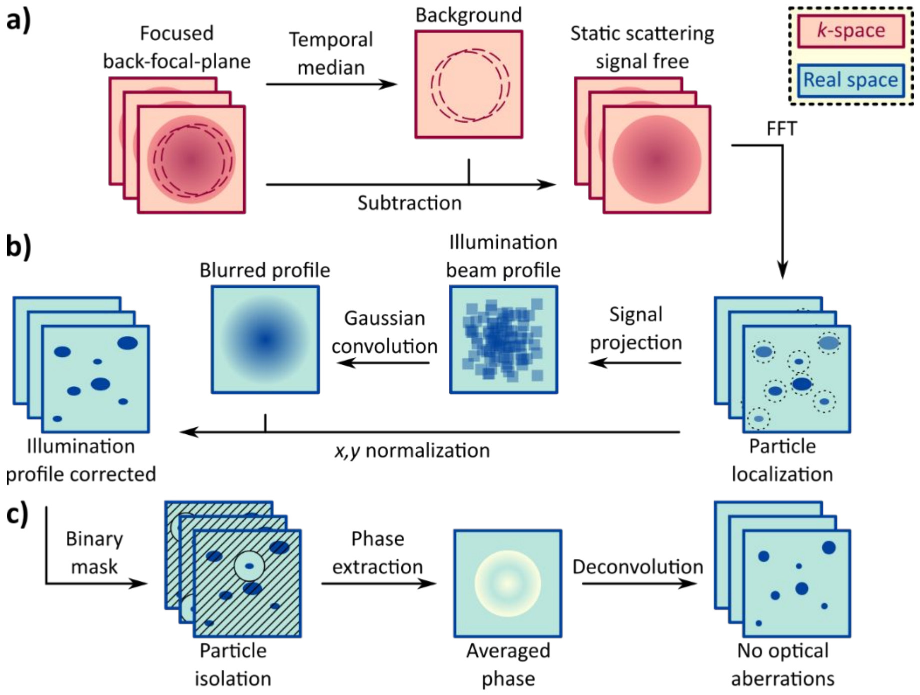

Figure S3, **Background and aberration correction.** A background of the static scattering signals is created by applying a temporal median to the previously processed images and subtracted from them. Images are transformed into the real-space and all the particles are localized. Illumination beam profile is reconstructed by projecting particle signals into a new image and convoluting it with a Gaussian function. Real-space imaged are divided by the resulting beam profile. Particles are isolated and their phase extracted. Images are deconvoluted by the average of the extracted phases, yielding on optical aberration-free images. Salmon colour refers to k-space while turquoise indicates real space.

The aberration corrected holograms are propagated along the optical axis according to the angular spectrum method (Kim, 2011). With that, we reconstruct several planes of a 3D volume from a 2D hologram (Figure S3). Specifically, the processed $M \times M$ pixel^2 *k*-space holograms are multiplied by the propagation kernel $\boldsymbol{K}$ and subsequently inverse Fourier transformed. Specifically, the propagation kernel has the form:

$$\boldsymbol{K}(x,y,z) = \exp\left(-i\sqrt{k_m^2 - k_x^2 - k_y^2}\, z\right)$$

where $k_m = 2\pi n/\lambda$, with n being the refractive index of medium through which the light propagates, corresponding to water in this work. The discretised spatial frequencies are $(k_x, k_y) = 2\pi(x,y)/M\Delta x$ for $(-M/2 \leq x,\ y \leq M/2)$ and with Δx representing the magnified pixel size of the imaging system. Representative planes obtained from a single hologram are shown in Figure 2b.

Figure S4 shows how 3D localisation is performed. Each hologram is first propagated from −40 µm up to +40 µm with a coarse spacing between different Z-planes (Δz) of 400 nm. The resulting 3D intensity maps are then segmented into regions of interests based on local maxima and Z-range set to 50-80 µm depending on the experiment. To achieve sub-pixel localisation, the particle-containing segmented regions of interest are propagated with a finer Δz spacing of 100 nm over a total of +/-2 µm with respect to their local maxima. We then determine the particles' maxima within a 1 µm x 1 µm region around the centre-of-mass and subsequently fit a parabola using the two most adjacent Z-pixel values along the maximum. For subpixel localisation along the XY-plane, particles that are in focus at the calculated

Z-plane are fitted using the radial centre symmetry algorithm (Parthasarathy, 2012). We extract particles' amplitude estimates. Finally, we follow the adaptive tracking algorithm of Jaqaman *et al.* to link all the 3D localisations and generate 3D tracks (Jaqaman et al., 2008). Only tracks longer than 100 time points are used for further analysis.

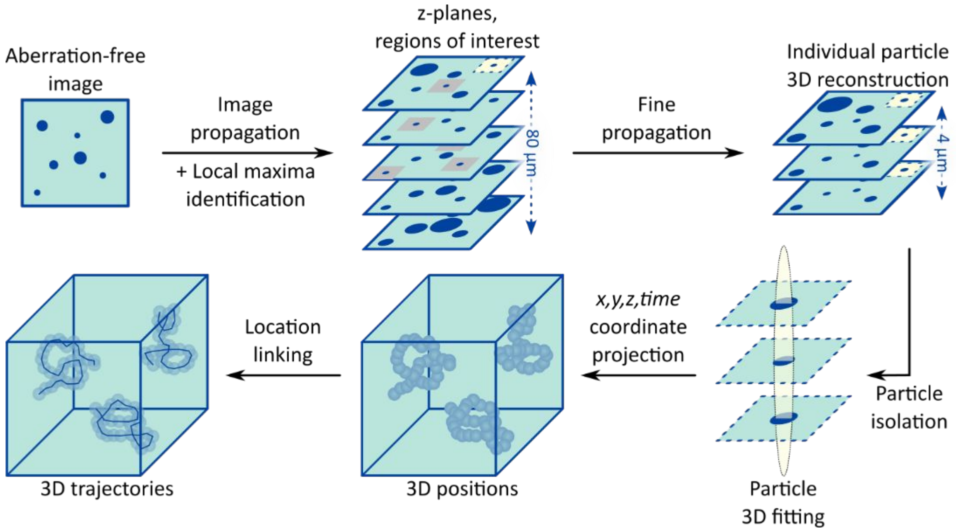

Figure S4, **Hologram propagation and 3D single particle tracking.** Aberration-free images are propagated. Regions of interest are identified based on local maxima (grey). A fine propagation is done for each plane containing said regions. Particles are individually isolated and their 3D signal fitted. Sample volume is reconstructed by projecting particle spatial and temporal positions. 3D trajectories are extracted by linking particle localizations overtime.
